# Food-related coping strategies against food insecurity among households in selected communities in South-East Nigeria

**DOI:** 10.1186/s40795-026-01266-8

**Published:** 2026-02-06

**Authors:** G. O. Iheme, C. R. Ajuluchukwu, E. M. Okonkwo, A. C. Amaeze, H. C. Ezenwa, U. C. Akpasuo, C. A Onyeagoro, O. C. Nzeagwu

**Affiliations:** 1 Department of Food Studies, Nutrition and Dietetics, Uppsala University, Uppsala, SE-751 22 Sweden; 2 Department of Human Nutrition and Dietetics, Michael Okpara University of Agriculture, Umudike, Nigeria

**Keywords:** Food insecurity, Food availability, Coping strategies, Households, Nigeria

## Abstract

**Introduction:**

Due to the heightened food insecurity levels experienced in low-income settings, many households resort to adopt various coping strategies. This study assessed the household food insecurity status and coping strategies in selected communities in South East Nigeria.

**Methods:**

A cross-sectional study design and multi-stage sampling technique were used to select 1244 households from six local government areas in the municipal city centres of Abia and Imo State. The Household Food Insecurity Access Scale (HFIAS) and CARE/World Food Program Coping Strategy Index protocol were used to collect data on food security and food-related coping mechanisms, respectively. IBM SPSS version 25.0 was used to analyse the data. Descriptive and inferential statistics were calculated, with p-values of < 0.05 considered statistically significant.

**Results:**

The findings indicated that more than half of the respondents were occasionally or frequently concerned about food (59.8%), unable to eat their favourite foods (57.3%), subject to food restrictions (55.5%), consumed unwanted foods (54.4%), reduced meal portions (57.2%), and ate fewer meals (55.1%) as a result of lack of resources or money. This culminated in the severe, moderate and mild food insecurity levels among 15%, 48.2% and 24.0% of the respondents. These food insecurity level have prompted the frequent adoption of dietary modifications and food restrictions as coping mechanisms. Coping strategies and food insecurity were significantly correlated (*p* < 0.01).

**Conclusion:**

The majority of households used coping mechanisms that reduced the quantity and quality of their food, as food insecurity was widespread. This highlights the need for targeted interventions to improve food security and strengthen household resilience to adopt safer and more sustainable coping strategies under prevailing circumstances.

## Introduction

Food security has emerged as a critical global policy concern, as the global rise in food prices, among other factors, negatively impacts access to food [1–3]. Food security is widely recognized as achieved when all individuals consistently have physical, social, and economic access to sufficient, safe, and nutritious food that meets their dietary requirements and personal preferences, thereby enabling them to lead active and healthy lives [4]. Approximately 2.3 billion individuals worldwide are estimated to have experienced moderate or severe food insecurity in 2024 [5]. The global prevalence of moderate or severe food insecurity has shown a slight decline since 2021, decreasing from 29.3 to 28.0% in 2024 [6]. Although the prevalence of food insecurity has exhibited a downward trend since 2021, a considerable number of individuals continue to experience hunger, with projections of global chronic undernourishment among over 500 million people in the world [6, 7].

Nigeria’s dire economic situation has severely worsened the nutritional status of its citizens, with over 31.8 million people facing acute food insecurity by the last quarter of 2024, particularly impacting vulnerable groups such as newborns, young children, pregnant and breastfeeding women, and low-income communities [8]. This crisis, marked by significant dietary challenges like protein-energy malnutrition and micronutrient deficiencies, has been exacerbated by soaring food prices driven by the removal of fuel subsidies, ongoing security threats from Boko Haram terrorism, banditry, and the farmers-herders conflict in Nigeria’s key agricultural regions, further compounding malnutrition among women and children [8]. Low-income countries and communities, including those in Nigeria, bear the brunt of hunger, food insecurity, and malnutrition, as they are disproportionately affected by food price inflation [6]. In these settings, poorer households, which allocate a larger share of their income to food, are particularly vulnerable to even slight price increases. Between 2019 and 2024, significant price surges in key diets like starchy staples and oils were observed in Nigeria, further jeopardizing food security and nutritional outcomes among economically disadvantaged groups [6]. While food insecurity levels appear relatively high in rural areas than urban settings, urban areas in low-income countries are still heavily affected by food insecurity [6]. Contextual factors associated with urbanization such as limited land availability for food production, rural-to-urban migration, increased time away from home due to formal employment, and shifting lifestyles underscore the importance of assessing food insecurity in urban populations [9–11].

In 2025, global initiatives to address food insecurity have demonstrated modest advancements; however, substantial obstacles persist, particularly in regions such as Sub-Saharan Africa and South Asia, as reported by the Food and Agriculture Organisation [7]. Thus, households within these regions are forced to adopt food-related coping strategies. Conventionally, the capacity of households to endure food insecurity has been defined by a range of behavioural adaptations that manifest as coping mechanisms, potentially highlighting household vulnerabilities based on socioeconomic and cultural influences [12]. Addressing food insecurity is a complex challenge, as urban households that face limited access to land for food production have to purchase most of the food they consume [13]. Previous studies showed that the strategies employed by low-income urban households can be categorized into two main types, which are direct strategies employed to adjust current food consumption practices in the light of food shortage or non-food-based based referring to other approaches to acquire money or resources needed for obtaining food [14–16]. Common adaptive food-based responses during periods of food scarcity, particularly in the context of individual-level crises such as escalating food prices, include purchasing less preferred food items, reducing meal portion sizes, relying on staple foods like rice, forgoing meals while liquidating assets, labour adjustments and reduced budgetary allocation for non-food items represents common examples of non-food based coping strategies [14–16].

Several studies have either compared composite indicators of food insecurity and coping strategy against various determinants or predictors [17, 18], or explored how overall food insecurity scores compare with their weighted coping strategies [19]. However, disaggregating household food security to explore its association with specific coping strategy items may offer new insights. Therefore, understanding these household-level coping mechanisms and their connection to food insecurity is essential for developing and implementing effective policies and programs to address them. This study examined the food-related coping strategies adopted by urban households in South-Eastern Nigeria in relation to their experiences of food insecurity.

## Methods

### Study design

This study employed a community-based cross-sectional design to examine food insecurity levels and the corresponding food-related coping strategies of urban households in Nigeria. This study was implemented across major municipal centres of Abia and Imo States, reflecting the urban variations in the assessed parameters.

### Population and sampling

A multi-stage sampling technique was employed in selecting the respondents. Firstly, because the study focused on urban households, Local Government Areas (LGAs) that constitute or closely align with municipal city centres of Abia and Imo State were purposively selected.

To ensure adequate representation across the major urban/peri-urban strata of the two state capitals, three LGAs were selected from each state.

Abia State; Umuahia North, Umuahia South and Ikwuano.

Imo State; Owerri Municipal, Owerri North and Owerri West.

Each selected L.G.A comprise 10–12 wards, only wards which are predominantly urban or sub-urban were included in the sampling frame for possible selection, with variations in the number of eligible wards dependent on their level of urbanization. For instance, while only four [8] wards in Ikwuano LGA were included, all the eleven [20] wards in Owerri municipal met the inclusion criteria.

Furthermore, two wards were selected per LGA using simple random sampling (balloting without replacement). In the final stage, a range of 100–105 participants were drawn from each selected ward using systematic household sampling. Details of the sampling frame and enlisted wards are available in Table 1.

**Table 1:** Study sampling frame

Eligible wards included in the sampling frame	Selected wards
Umuahia North	
Ibeku East I; Ibeku East II; Ndume; Ibeku West; Umuahia Urban I; Umuahia Urban II; Umuahia Urban III, Afugiri	Afugiri and Ibeku East II
Umuahia South	
Ezeleke / Ogbodiukwu; Old Umuahia; Amakama; Ubakala ‘A’; Ubakala ‘B’	Amakama and Ubakala B
Ikwuano	
Ibere 1, Ibere II, Oboro I, Oboro II	Ibere II, Oboro II
Owerri Municipal	
Aladinma I, Aladinma II, Ikenegbu I, Ikenegbu II, Azuzu I, Azuzu II, Azuzu III, Azuzu IV, GRA, New Owerri I, New Owerri II	Ikenegbu II, New Owerri I
Owerri North	
Awaka / Ihitte-Ogada; Naze; Egbu; Orji; Ihita-Oha; Obibi-Uratta I; Obibi-Uratta II	Naze, Orji
Owerri West	
Avu / Oforola; Umuguma; Eziobodo; Ihiagwa; Nekede; Obinze	Nekede, Obinze

### Data collection

Data was collected between July and November 2023 using a structured questionnaire. This questionnaire was either self-administered or delivered in an interviewer-assisted approach, depending on the participant´s literacy level. In both cases, a trained research assistant supported the household head or spouse in completing the instrument. Through this technique, responses on the household’s socioeconomic characteristics, food security and food-related coping mechanisms were elicited. Details of the development and validation of these widely adopted study instruments have been reported elsewhere [20–22].

Any available household heads or spouses who demonstrated a willingness to participate in the study were recruited. Children, adolescents, unmarried individuals living alone, cohabiting non-family members, and other dependents were excluded from the study. The focus was on adults with primary decision-making responsibility for household food acquisition, provision and preparation.

### Data analysis

#### Food coping strategy (FCS)

The food coping strategies adopted by families were determined using the Coping Strategy Index [20], which indirectly examines the adaptive behaviour utilized to mitigate food insecurity risks. The coping strategy consists of fourteen questions that assess the rate and extent of household responses to food insecurity, encompassing practices associated with dietary changes, measures to increase food availability or reduce the number of people to feed, and various rationing strategies, over a 7-day interval [20]. However, the severity ranking was adapted to reflect the extent of each coping behaviour, ranging from “*none*” (assigned a score of 0) to “*often”* (assigned a score of 4).

#### Household food insecurity access scale (HFIAS)

Household food insecurity was measured using the HFIAS tool developed by the Food and Nutrition Technical Assistance Project [21]. This tool comprises nine questions on the status and frequency of occurrence of food insecurity-related behaviour among households over a four-week duration. While a score of 0 is assigned to “Never” responses to the occurrence, frequency of occurrence was assigned accordingly; 1 (rarely: once or twice), 2 (sometimes: three to ten times), or 3 (often: more than ten times) [22].

Furthermore, following the standard HFIAS algorithm for food insecurity categorization, households were categorized as food secure (depicting minimal or no worry about food), mildly food insecure (characterized by occasional worry or reliance on less preferred food alternatives with no quantity reduction), moderately food insecurity (demonstrated by substantial reductions in food quality and quantity) and severely food insecure (persistent food reductions potentially leading to complete food absence for a specific period or even an entire day) [22].

### Statistical analysis

Descriptive statistics (frequency, percentage, mean and standard deviation) were computed for both continuous and categorical variables to summarize household socioeconomic characteristics, food security status, and food-related coping strategies.

Given the study´s focus to examine how individual food insecurity elements are connected to the distinct coping strategies and the ordinal nature of the variables, Spearman´s rank correlation was used, with *p* < 0.05 judged as statistically significant.

## Results

### Individual and household characteristics

Information on the individual and household characteristics of the study population is presented in Table 2. Results showed that household heads were predominantly males (78.4%), while females accounted for 21.6%. Majority, (70.4%) of the participants were currently married, whereas 17.5% were widowed and 7.9% were divorced. Almost all (94.4%) of them identified as Christians. A substantial proportion of the respondents had a tertiary educational qualification, ranging from 17.6% with diplomas or related certifications to 23.5% with university degrees or advanced qualifications, this was closely by those with secondary (30.1%) and primary (24.1%) education qualification. About a quarter (26.3%) of the respondents earned between ₦30,000 and ₦50,000 (≈$40–60), while a similar proportion either earned less than ₦30,000 (<≈$40) (29.6%) or between ₦50,000 and ₦70,000 (≈$60–91) (28.9%). Access to basic household amenities, such as a water-cistern toilet (75.7%), tap water (62.5%), and living in 1-3-bedroom flats (67.1%), was common.

**Table 2:** Personal and household characteristics

Variables	Frequency (*n* = 1244)	Percentage (%)
Head of household		
Female	269	21.6
Male	975	78.4
Marital status		
Married	876	70.4
Divorced	98	7.9
Widowed	218	17.5
Single	52	4.2
Religion		
Christianity	1174	94.4
Islam	8	0.6
Traditional religion	62	5.0
Household head highest education		
No formal education	55	4.4
Primary school	300	24.1
Secondary school	374	30.1
Tertiary education 1 (Sub-degree; OND, SRN, TIC)	219	17.6
Tertiary education 2 (Degree; HND, BSc, MSc, PHD)	296	23.8
Toilet used in household		
Water Cistern	942	75.7
Pit latrine	302	24.3
Household water supply		
Tap	777	62.5
Well	83	6.7
Stream/River	46	3.7
Community borehole	329	26.4
Apartment type		
Single room	119	9.6
One room self-contained	90	7.2
One-bedroom flat	135	10.8
2–3 bedroom flat	328	26.4
Bungalow	506	40.7
Duplex/mansion	66	5.3
Estimated household income		
< ₦30,000 (<≈$40)	368	29.6
₦30,000–50,000 (≈$40–60)	327	26.3
₦51,000–70,000 (≈$60–91)	154	12.4
₦71,00-100,000 (≈$92–130)	205	16.5
>₦100,000 (>$130)	182	14.6
Preferred not to say	8	0.6

### Household food security level

Table 3 presents the household food insecurity levels of respondents. Findings indicate that the proportion of households reporting “never” generally decreased as the questions reflected more severe forms of food insecurity; worry about food (14.6%), eating less preferred foods (18.7%), consuming fewer varieties of food (22.6%), eating meals they did not want (24.2%), reducing meal portions (21.0%), eating fewer meals per day (21.5%), having no food available in the household (41.3%), going to bed without eating (50.6%), and spending an entire day without food (60.6%).

This translated to more than half of the households being occasionally or frequently concerned with, or engaging in, certain food-related restrictions, while a smaller proportion (15.7–27.0%) reported a complete absence of foods for specific periods.

**Table 3:** Participant’s response to household food insecurity and access scale questions

Food insecurity assess scale response	Never F (%)	Rarely F (%)	Sometimes F (%)	Often F (%)
Worry about food	182 (14.6)	318(25.6)	452 (36.3)	292 (23.5)
Unable to eat preferred foods	233 (18.7)	299 (24.0)	485 (39.0)	227 (18.2)
Eat just a few kinds of foods	281 (22.6)	273 (21.9)	481 (38.7)	209 (16.8)
Eat foods they do not want to eat	301 (24.2)	267 (21.5)	445 (35.8)	231 (18.6)
Consume smaller meals	261 (21.0)	271 (21.8)	514 (41.3)	198(15.9)
Consume fewer meals	268 (21.5)	223.3)	484 (38.9)	201 (16.2)
No any kind of food in the house	514 (41.3)	394 (31.7)	223(17.9)	113 (9.1)
Go to sleep hungry without eating	629 (50.6)	375 (30.1)	165 (13.3)	75 (6.0)
Stay a whole day and night without food	754 (60.6)	294 (23.6)	135 (10.9)	60 (4.8)

### Categorised food security status of households

**Fig. 1:**
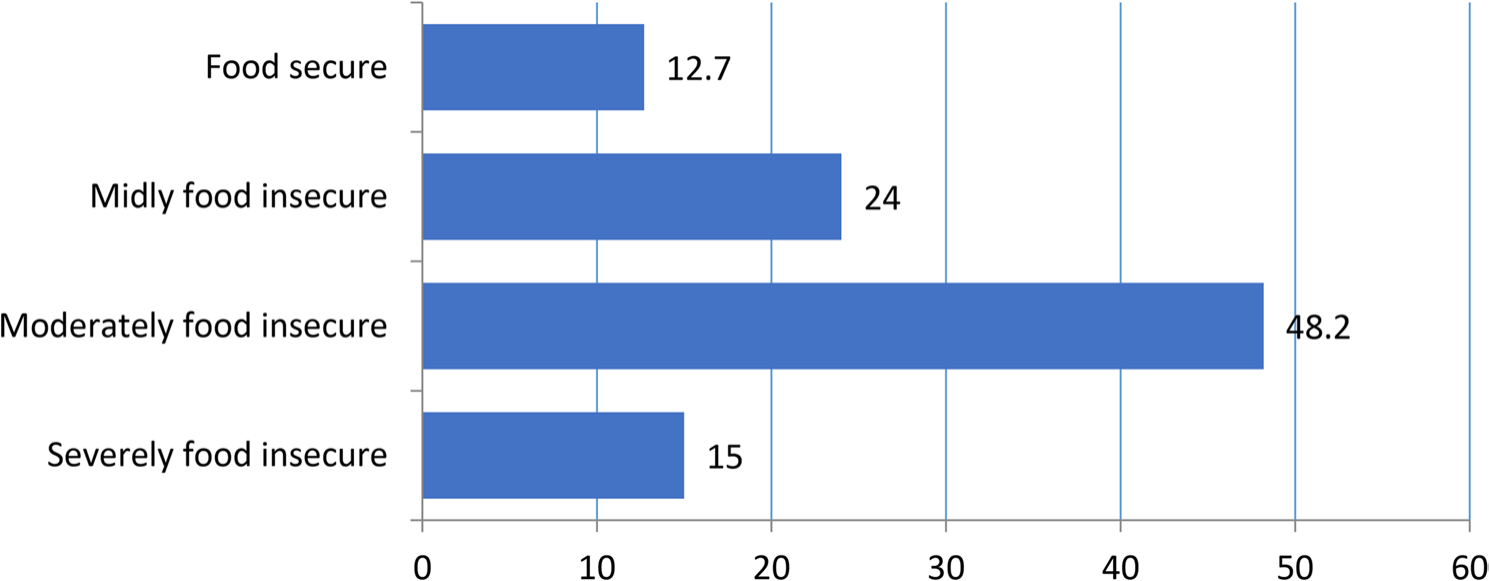
Categorised food security status of households

Figure 1 shows the categorised food security status of selected households. The Majority (87.3%) of the households reported experiencing food insecurity, while only 12.7% of them were food secure. Among food-insecure households, most fell within the mild (24.0%) and moderate (48.2%) categories, followed by severe food insecurity (15.0%).

### Participant’s response to food coping strategies

Information on the respondents’ coping strategies is summarized in Table 4. More than 10% of the households reported always employing the following coping strategies; relying on less preferred (12.2%) or less expensive foods (16.5%), purchasing food on credit (10.7%) or using borrowed money (10.7%), reducing meal frequency (12.8%), decreasing portion size (13.8%), and limiting adult food intake to prioritize children (10.1%). All except one of these strategies - limiting adult food intake to prioritise children were occasionally applied by approximately one-third (31.1–38.9%) of the respondents. Overall, the least adopted coping strategies were sending household members to eat elsewhere (sometimes: 13.8%; often: 6.9%), sending them to beg (sometimes: 19.9%; often: 8.9%), gathering wild or immature foods (sometimes: 16.6%; often: 6.7%), and skipping meals for an entire day (sometimes: 19.4%; often: 8.0%). 

**Table 4:** Participants’ response to food coping strategies

S/*N*	Coping strategies	None F (%)	Rarely F (%)	Sometimes F (%)	Often F (%)
1	Rely on less preferred	208(18.4)	358 (31.7)	425 (37.6)	138 (12.2)
2	Rely on less expensive food	325(28.0)	199 (17.1)	446 (38.4)	192 (16.5)
3	Borrow money to buy foodstuffs	325(34.9)	216 (23.2)	290 (31.1)	100 (10.7)
4	Purchase food on credit	225(23.1)	230 (23.6)	414 (42.5)	104 (10.7)
5	Rely on help from friends or relatives	370(40.6)	170 (18.7)	288(31.6)	83 (9.1)
6	Limit portion size at mealtimes	311(28.9)	198 (18.4)	419 (38.9)	148 (13.8)
7	Limit adult intake	394(39.0)	208 (20.6)	308 (30.5)	96 (9.5)
8	Reduce the number of meals eaten in a day	330(31.5)	218 (20.8)	364 (34.8)	134 (12.8)
9	Skip the whole day without eating	428(47.8)	222 (24.8)	174 (19.4)	72 (8.0)
10	Gather wild food, hunt or harvest immature crops	427(48.9)	190(21.5)	207 (16.6)	59 (6.7)
11	Consume seed stocks held for next season	396(44.2)	225 (25.1)	202 (22.6)	72 (8.0)
12	Send household members to eat elsewhere	430(48.9)	191 (15.3)	172 (13.8)	86 (6.9)
13	Send household members to beg	434(49.9)	186 (21.4)	173 (19.9)	77 (8.9)
14	Restrict adult’s intake for small children to eat	410(44.5)	168 (18.2)	250 (27.1)	93 (10.1)

### Relationship between food insecurity and food coping strategy of participants

The connection between food coping strategies and food insecurity is presented in Table 5. The findings showed that all of the responses on the food insecurity access scale and the food coping strategies had a substantial (*p* < 0.01) positive relationship. The implication of r (0.10–0.63) in this finding is that as the severity of individual food insecurity indicators progressed from worrying about food to experiencing an entire day or night without food, the intensity of coping strategies also increased from reliance on less preferred/expensive foods to sending the household to beg or eat elsewhere.

**Table 5:** Relationship between food insecurity and coping strategies of participants and the food coping strategy of participants

Household Food Insecurity Access Scale variables	Worry about food	Unable to eat preferred food	Eat just a few kinds of foods	Eat foods they do not want to eat	Reduce the frequency of food	Reduce the number of meals	No kind of food in the house	Go to sleep hungry without eating	Stay a whole day and night without food	Overall HFIAS score
Coping Strategies Variables										
Rely on less preferred	0.505^**^	0.596^**^	0.548^**^	0.560^**^	0.540^**^	0.529^**^	0.377^**^	0.408^**^	0.359^**^	0.375^**^
Rely on less expensive food	0.373^**^	0.427^**^	0.398^**^	0.447^**^	0.360^**^	0.355^**^	0.268^**^	0.424^**^	0.390^**^	0.260^**^
Borrow money to buy foodstuff	0.288^**^	0.424^**^	0.489^**^	0.527^**^	0.471^**^	0.477^**^	0.507^**^	0.536^**^	0.556^**^	0.359^**^
Purchase food on credit	0.329^**^	0.453^**^	0.514^**^	0.536^**^	0.512^**^	0.527^**^	0.466^**^	0.485^**^	0.453^**^	0.357^**^
Rely on help from friends or relative	0.192^**^	0.328^**^	0.390^**^	0.443^**^	0.380^**^	0.389^**^	0.469^**^	0.472^**^	0.531^**^	0.294^**^
Limit portion size at mealtimes	0.311^**^	0.410^**^	0.466^**^	0.501^**^	0.465^**^	0.461^**^	0.354^**^	0.435^**^	0.407^**^	0.343^**^
Limit adult intake	0.144^**^	0.211^**^	0.250^**^	0.284^**^	0.269^**^	0.267^**^	0.239^**^	0.293^**^	0.290^**^	0.192^**^
Reduce the number of meals eaten in a day	0.297^**^	0.396^**^	0.458^**^	0.498^**^	0.466^**^	0.475^**^	0.351^**^	0.438^**^	0.417^**^	0.304^**^
Skip the whole day without eating	0.119^**^	0.260^**^	0.307^**^	0.373^**^	0.316^**^	0.322^**^	0.512^**^	0.550^**^	0.681^**^	0.273^**^
Gather wild food, hunt or harvest immature crops	0.102^**^	0.244^**^	0.302^**^	0.365^**^	0.304^**^	0.312^**^	0.401^**^	0.439^**^	0.532^**^	0.242^**^
Consume seed stocks held for next season	0.109^**^	0.255^**^	0.308^**^	0.368^**^	0.309^**^	0.321^**^	0.473^**^	0.494^**^	0.576^**^	0.289^**^
Send household members to eat elsewhere	0.106^**^	0.261^**^	0.314^**^	0.373^**^	0.307^**^	0.325^**^	0.499^**^	0.539^**^	0.632^**^	0.259^**^
Send household members to beg	0.07^**^	0.230^**^	0.282^**^	0.348^**^	0.283^**^	0.294^**^	0.479^**^	0.510^**^	0.619^**^	0.240^**^
Restrict adult’s intake for small children to eat	0.153^**^	0.292^**^	0.346^**^	0.414^**^	0.353^**^	0.362^**^	0.421^**^	0.494^**^	0.548^**^	0.273^**^

^**^ Statistically significant at *P* < 0.01

## Discussion

Millions of people and families are today affected by food insecurity, which is a concerning problem worldwide, particularly in developing nations. In line with our study, recent global evidence continues to spotlight Nigeria as a home to one of the most food-insecure populations in the world [6].

The socio-economic variables, which presented a preponderance of male-dominated household-heads and Christian affiliation, are consistent with reports from south-east Nigeria, which also highlighted the prevalence of these characteristics [23, 24]. Although most respondents in this study had at least a secondary school education, access to basic amenities was common despite low-income levels In contrast, evidence suggests that higher education generally improves a household’s financial status and living conditions, which can in turn enhance food security [25–27]. However, in municipal settings, basic amenities are generally more readily available regardless of socioeconomic status, which may explain this pattern. On the other hand, educated individuals are likely to attract white-collar jobs situated in favourable locations, thus invariably influencing the housing quality of nearby residential areas [28]. Nevertheless, the nation´s economic challenges will impact on the limited earning capacity of the studied households and compromise food security, as food prices skyrocket and purchasing power drops considerably [29, 30].

The high food insecurity level reported in this study compares closely with a study by Ukonu et al., [31] conducted within this region, while a similar study reported lower food insecurity levels (61.0-63.3%) [32, 33]. This finding contrasts with the widely documented global pattern in which food insecurity is more prevalent among rural populations, especially since some comparable studies report results based on mixed rural–urban samples [6]. Therefore, addressing the contextual factors surrounding food insecurity level is critical given its wider public health implications. Food insecurity is associated with increased levels of malnutrition, particularly among vulnerable groups [34, 35].

Reliance on coping mechanisms as reported in this study is linked to less food availability in literature [36]. High dependence on less expensive foods in this study compares well with findings from Akerele et al. [37], which acknowledged this as a common approach used by rural households in Southwest Nigeria. Food-related rationings in terms of reduction in portion size or meal number were key coping mechanisms adopted in this study, and this aligns as common coping strategies reportedly used by Nigerian households during persistent food shortages [38, 39]. The rising food inflation due to the nation’s present economic downturn continues to influence food-related decisions with cost emerging as a significant determinant [30, 40].

Similar to the present study, several studies have demonstrated that higher coping strategy use corresponds with worsening household food availability, whether reflected through overall scores or along the severity continuum of individual items [19, 20, 41, 42]. This pattern further underscores the internal validity of both the HFIAS and the Coping Strategy Index (CSI) in capturing the progression of food insecurity.

## Conclusion

This study revealed a high prevalence of food insecurity ranging from mild to severe among selected households. While the most common coping strategies involved financial adjustments and food rationing, the severity of individual food insecurity items was consistently associated with corresponding coping strategies, though with varying levels of intensity.

Addressing this urgent challenge requires coordinated, multi-sectoral interventions targeted at expanding access to affordable, nutrient-dense foods in disadvantaged areas, strengthening social safety nets and assistance programs for low-income households, and promoting agricultural initiatives, job training, and financial literacy are critical steps. Effective responses will also demand collaboration across government, community, and development stakeholders to improve household resilience and advance food and nutrition security in the region.

## Data Availability

Upon request, the corresponding author will provide the datasets for this study.
